# The osteoblast as an inflammatory cell: production of cytokines in response to bacteria and components of bacterial biofilms

**DOI:** 10.1186/s12891-016-1091-y

**Published:** 2016-06-02

**Authors:** Ulrike Dapunt, Thomas Giese, Sabine Stegmaier, Arash Moghaddam, Gertrud Maria Hänsch

**Affiliations:** Center for Orthopaedics, Trauma Surgery and Spinal Cord Injury, Heidelberg University Hospital, Schlierbacher Landstrasse 200a, Heidelberg, 69118 Germany; Institute for Immunology, Heidelberg University, Im Neuenheimer Feld 305, Heidelberg, 69120 Germany; HTRG Heidelberg Trauma Research Group, Center for Orthopaedics, Trauma Surgery and Spinal Cord Injury, Heidelberg University Hospital, Schlierbacher Landstrasse 200a, Heidelberg, 69118 Germany

**Keywords:** Osteoblast, Biofilm infection, GroEL, Extracellular polymeric substance

## Abstract

**Background:**

Implant infections are a major complication in the field of orthopaedics. Bacteria attach to the implant-surface and form biofilm-colonies which makes them difficult to treat. Not only immune cells exclusively respond to bacterial challenges, but also local tissue cells are capable of participating in defense mechanisms. The aim of this study was to evaluate the role of osteoblasts in the context of implant infections.

**Methods:**

Primary osteoblasts were cultivated and stimulated with free-swimming bacteria at 4 °C and 37 °C. Supernatants were harvested for ELISA and expression of pro-inflammatory cytokines evaluated by RT-PCR. Bacterial binding to osteoblasts was evaluated using cytofluorometry and uptake was investigated by ^3^H thymidine-labelling of bacteria. Osteoblasts were additionally stimulated with the extracellular polymeric substance (EPS) of *Staphylococcus epidermidis* biofilms, as well as components of the EPS; the bacterial heat shock protein GroEL in particular.

**Results:**

We demonstrated that binding of bacteria to the osteoblast cell surface leads to an increased production of pro-inflammatory cytokines. Bacteria are capable of surviving intracellular. Furthermore, osteoblasts do not only respond to free-swimming, planktonic bacteria, but also to components of the EPS, including lipoteichoic acid and the heat shock protein GroEL.

**Conclusion:**

In conclusion, local tissue cells, specifically osteoblasts, might contribute to the persistence of the inflammatory response associated with implant-infections.

## Background

Osteoblasts are primarily studied in the context of bone turnover and fracture healing. However, they can also acquire characteristics of inflammatory cells. For example, in response to bacterial challenge synthesis and release of pro-inflammatory cytokines is described, including generation of CXCL8 (interleukin 8, IL-8) or CCL2 (monocyte chemoattractant protein-1, MCP-1). Because both, CXCL8 and CCL2, attract leukocytes, the local inflammatory response might escalade [1–4] (for review see [5, 6]). Of note, osteoblasts also acquire properties of antigen presenting cells. Following uptake of *Staphylococcus aureus*, expression of MHC class II molecules and of co-stimulatory receptors was described [7, 8], as was the ability of MHC class II positive osteoblasts to present superantigens to T lymphocytes. Furthermore, osteoblasts produce defensin, a molecule with bactericidal activity that was initially described in leukocytes [9].

Osteoblasts take up bacteria by an active process [10, 11]. Whether this uptake is a host defence reaction, analogous to the bacteriophagocytosis by leukocytes is still an open question. Under experimental conditions, ingested bacteria survive within the cell, leading to the notion that the osteoblast might provide a safe hiding place, which - in consequence - might contribute to the persistence of an infection [12–16].

Interactions of bacteria, either with phagocytic cells or osteoblasts, are mainly studied with free-swimming, planktonic bacteria. However, this is not the only life-style of bacteria, and possibly not even the preferred one, which was recognised over the last few years. Rather, many bacteria species live in structured colonies, embedded in an extracellular polymeric substance, so-called “biofilms” [17–20]. By means of biofilm formation, opportunistic bacteria in particular may acquire a pathogenic potential.

Our research group is particularly interested in infections due to biofilm formation on orthopaedic implants. In these infections, staphylococci species are prevalent [21]. Extensive inflammation around the implant occurs, which eventually results in bone degradation and in loosening of the prosthesis. Analysis of the local host response revealed infiltration of phagocytic cells, particularly of neutrophils, generation of pro-inflammatory cytokines and increased development of bone-resorbing osteoclasts [22–24].

A likely source for these cytokines are infiltrating neutrophils, which are activated by bacterial biofilms. Of note, entities within the extracellular substance of the biofilm activate neutrophils, among those peptidoglycan, lipoteichoic acid and the bacterial heat shock protein GroEL [25, 26]. Another source could be osteoblasts, which are also known to produce cytokines after appropriate stimulation [3–5]. We now addressed the question whether osteoblasts are not only activated by planktonic staphylococci, but by entities derived from bacterial biofilms.

## Methods

### Culture of osteoblasts

Primary osteoblasts were cultivated from human bone marrow which was harvested either from the femoral bone using the RIA (reamer-irrigator-aspirator)-technique or from the iliac crest of patients undergoing surgery due to fracture malunion or non-union, and who required an autologous bone graft. Informed consent was obtained from the patients, and the study was approved by the local ethic committee of Heidelberg University (S-355/2010; October 28th 2010). Samples were grinded using sterile scalpels and cultivated in osteoblast growth medium (PromoCell, Heidelberg, Germany) containing 0.1 % penicillin/streptomycin (Gibco Life Technologies, Eggenstein, Germany). Outgrowth of cells occurred usually between 4 to 8 days. Cells were subcultivated following digestion with trypsin (0.05 % Trypsin-EDTA, Life Technologies) for 5 min at 37 °C and resuspended in osteoblast growth medium. After 10 to 14 days, homogenous cell layers were seen; osteoblasts were identified by expression of collagen type I and bone sialoglycoprotein, and lack of markers for myeloid cells (CD11b, CD68) (all from Beckman Coulter, Krefeld, Germany) as seen by laser scan microscopy and by cytofluorometry of detached cells. Expression of CD90 (Beckman Coulter, Krefeld, Germany) was used as a marker for de-differentiation. Osteoblasts were used for a maximum of two passages and experiments were carried out in 6 or 24-well dishes (NuncTM, Wiesbaden, Germany) at a concentration of 2 × 10^5^ cells/mL in osteoblast growth medium.

### Cytofluorometry and microscopy

Cytofluorometry was performed with paraformaldehyde-fixed osteoblasts using FACS Calibur and Cell quest pro as software (Becton and Dickinson, Heidelberg, Germany). Presence of collagen type I and bone sialoglycoprotein was assessed by indirect immunofluorescence. Slides were viewed by Laser scan microscopy (LSM, Leica).

### Stimulation of osteoblasts by bacteria

*Staphylococcus aureus* (Seattle 1945, ATCC 25923, Wesel, Germany) and *Staphylococcus epidermidis* (RP62a, ATCC 35984) were grown overnight on a blood agar plate at 37 °C (number PB5039A, Thermo Scientific, Germany, Wesel). The following day, bacteria were scraped of the plate, suspended in phosphate buffered saline and adjusted to 1x10^8^ cells/mL. Bacteria were added in a ratio of 1:20, 1:100 and 1:500 bacteria per osteoblast (2x10^5^ cells per well in a 6-well culture dish in a volume of 4 mL) and incubated at either 4 °C or 37 °C for the times indicated in the respective experiments (two different temperatures were compared, because phagocytosis is impaired at 4 °C). The supernatant was discarded and 1600 μg vancomycin/4 mL osteoblast growth medium was added for 30 min. The supernatant was discarded and replaced with 4 mL osteoblast growth medium containing 80 μg vancomycin and culture was continued for the times indicated in the respective experiment. In another set of experiments, heparin 200 μg (Heparin Sodium 25000, Ratiopharm, Ulm, Germany) was added before stimulation with bacteria and pre-incubated at room temperature for 10 min. After that, bacteria were added and experiments carried out as described above.

For RT-PCR analysis, cells were collected in 400 μL lysis buffer from the MagnaPure mRNA Isolation Kit I containing 1 % DTT (v/w) (ROCHE Applied Sciences - RAS, Mannheim, Germany). To determine release of cytokines, culture was continued for 6 h, 24 h and 48 h at 37 °C. Supernatants were then harvested and stored at−20 °C for ELISA (see below).

### Binding of bacteria to osteoblasts

FITC-labelled dead *S. aureus* were purchased (Molecular probes, S-2851, ThermoFisher Scientific, Schwerte, Germany). *S. epidermidis* were labelled with fluorescein isothiocyanate (FITC) according to the following protocol: FITC isomer I was purchased (Sigma- Aldrich, Darmstadt, Germany; F7250). A 5 mg/mL DMSO stock solution was diluted in phosphate buffered saline 1:10 (end concentration 0.5 mg/mL) and was incubated with bacteria (8x10^7^) for 45 min at 37 °C. Following repeated washing, the bacteria were suspended in 4 % formaldehyde. FITC-labelling of bacteria was evaluated by cytofluorometry. Binding of FITC-labeled bacteria to osteoblasts was determined by incubating with bacteria at a ratio of 1:100 bacteria per osteoblast for 2 h at either 37 °C or 4 °C, followed by cytofluorometry and by laser scan microscopy.

To visualise uptake of bacteria, osteoblasts were incubated with FITC-labelled bacteria (see below) at a ratio of 1:100 for 2 h. The cells were fixed and viewed by LSM.

### Uptake of ^3^H thymidine-labelled bacteria

An overnight culture of *S. epidermidis* in trypticase soy broth was performed. The next morning 2 mL of the overnight culture were added to 20 mL fresh trypticase soy broth containing 100 μL ^3^H Thymidin (Amersham, UK, TRA120 1 mCi/mL) and culture was continued for 4 h. Radioactivity associated with the bacteria was quantified. Osteoblasts were then incubated with the labelled bacteria as described above for 3 h. Then cell-associated radioactivity was measured in an aliquot, the remaining cells were treated with vancomycin to kill adherent, but not internalised cells (as described above), then lysed. An aliquot of the lysate was placed onto agar plates, and colonies were counted after 24 h.

### Formation of biofilms and extraction of the extracellular polymeric substance (EPS)

*S. epidermidis* was added to 1.5 L of pre-warmed Trypticase Soy Broth (TSB) to reach a final concentration of 3x10^6^ CFU/mL, then transferred to 30 polysterol dishes (Nunc 150x20, Thermo Fisher Scientific, Roskilde, Denmark) with a final volume of 50 mL per dish. After incubation for 2 days at 37 °C without shaking, the medium was removed and the remaining biofilm was scrapped off. The following treatment was adapted from Liu et al. (2002) [27]: per 10 mL of slime, 60 μL of 37 % formaldehyde was added and mixed for 1 h at 4 °C, followed by the addition of 4 mL 1 M NaCl and mixing for another 3 h at 4 °C. The resulting suspension was then centrifuged (Sorvall 5B Plus) for 15 min (18000 rpm at 4 °C). The pellet was discarded, the supernatant filtrated (Millex Syringe-driven Filter Unit 0.22um, Merck Milipore Ltd, Tullagreen, Ireland) and then dialysed overnight against Milipore water at 4 °C (membrane cut off 3600 Da; Spectrum Labs, Rancho Dominguez, CA, USA). The water was replaced and the isolated EPS was again dialysed for another 3 h, then concentrated using Vivaspin 20 (Sartorius Stedim Biotech, Göttingen, Germany) to a final volume of 4 mL and frozen at−20 °C until use. Extraction of EPS from 1.79 square meters biofilm yielded on average 44.9 mg of protein.

To detect possible endotoxin contamination a limulus assay was performed (Pierce LAL Chromogenic Endotoxin Quantitation Kit, Thermo Scientific, Bonn, Germany) following the instructions provided by the manufacturer. The adsorption of LPS was accomplished using Pierce High Capacity Endotoxin Removal Spin Column following the instructions provided but adjusting the incubation time to 2 h in order to maximize LPS-removal.

### Stimulation of osteoblasts

LTA (Sigma Aldrich) and recombinant GroEL (Enzo Life Sciences) were purchased. Osteoblasts were cultivated with LTA1 or LTA5 (1 μg/mL and 5 μg/mL, respectively), GroEL1 and GroEL5 (1 μg/mL and 5 μg/mL, respectively) or EPS2 and EPS10 (2 % and 10 % (v/v)). Supernatants were collected after 6 h and 24 h incubation time at 37 °C and cells were collected in lysis buffer for RT-PCR-analysis (see below).

### Quantitative real-time polymerase chain reaction

Cells were collected in 400 μL lysis buffer from the MagnaPure mRNA Isolation Kit I containing 1 % DTT (v/w) (ROCHE Applied Sciences - RAS, Mannheim). mRNA was isolated with the MagnaPure-LC device using the mRNA-I standard protocol. An aliquot was reversely transcribed using AMV-RT and oligo-(dT) as primer (First Strand cDNA synthesis kit, Roche) according to the manufactures protocol in a thermocycler. Primer sets optimized for the LightCycler® (RAS, Mannheim Germany) were developed and purchased from SEARCH-LC GmbH (www.Search-LC.com). The PCR was performed with the LightCycler® FastStart DNA Sybr GreenI kit (RAS) according to the protocol provided in the parameter specific kits. To control for specificity of the amplification products, a melting curve analysis was performed. The copy number was calculated from a standard curve, obtained by plotting known input concentrations of four different plasmids at log dilutions to the PCR-cycle number (CP) at which the detected fluorescence intensity reaches a fixed value. To correct for differences in the content of mRNA, the calculated transcript numbers were normalized according to the expression of the housekeeping gene peptidylprolyl isomerase B (PPIB). Values were thus given as transcripts per 1000 transcripts of PPIB.

### ELISA

IL-8, IL-6 and CCL2 in cell culture supernatants were determined using commercially available ELISA kits according to the protocol provided by the manufacturer. The human Elisa kits were purchased from R& D Systems (Minneapolis, USA).

### Statistical analysis

Differences between groups were calculated using ANOVA one-way or Mann–Whitney test using Origin 9.0 software. Significance level was determined as *P* < 0.05.

## Results

### Osteoblasts in culture

**Fig. 1:**
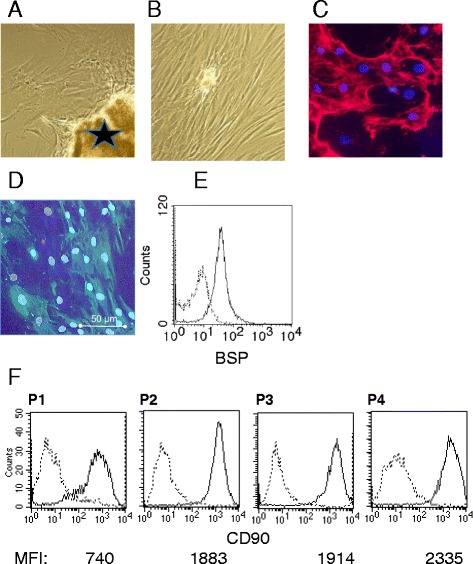
Culture and characterization of osteoblasts: Bone marrow was shredded and cultivated. **a** Osteoblasts started growing and after 10–14 days (the asterix indicates bone); **b** a near confluent layer of homogenous cells was obtained. **c** The cells produced collagen type I (stained in red; nuclei in blue); and **d** bone sialoglycoprotein (BSP) (stained in green) as seen by laser scan microscopy and **e** by cytofluorometry of detached cells (thick line; the thin line shows the isotype control). **f** The osteoblasts in early passage express CD90 (thick line; the thin line shows the isotype control); expression increases with time in culture and passage number as seen by an increase of the mean fluorescence intensity (MFI)

Numerous methods are available to raise osteoblasts from bone marrow. Under our culture conditions and with use of a special medium that prevented growth of fibroblasts, homogenous cultures were obtained within 10–14 days. Characteristic for osteoblasts is the production of type I collagen and of bone sialoglycoprotein (examples in Fig. 1a–e) and lack of markers for myeloid cells (CD11b, CD68) (data not shown). Under culture conditions, osteoblasts undergo a dedifferentiation, which can be followed up by the acquisition of the surface protein CD90, the expression of which increased steadily with time in culture, or number of passage, respectively (Fig. 1f). For our experiments we used cells in passage 1 and 2, only. Cells derived from different donors varied somewhat with regard to their response to bacterial challenges, which explains the wide variation seen in some of the experiments.

### Phagocytosis of bacteria and release of cytokines

**Fig. 2:**
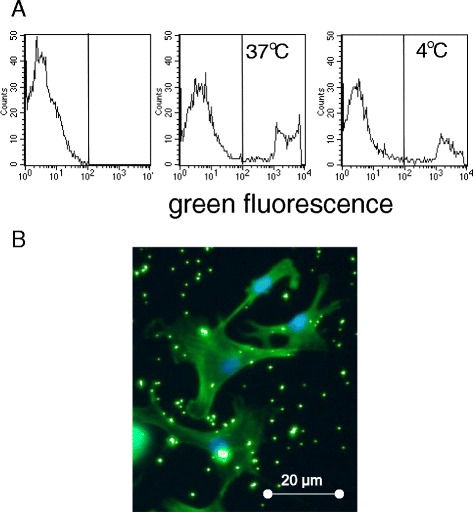
Binding and uptake of *S. aureus* to osteoblasts: **a** Osteoblasts were incubated with FITC-labelled bacteria at either 37 ° C or 4 °C. Then fluorescence associated with the cells was determined (the very left panel shows the autofluorescence of the cells). **b** Binding and uptake of bacteria could be confirmed by laser scan microscopy (bacteria in green; nuclei in blue)

To assess binding and uptake of bacteria, osteoblasts were cultivated with FITC-labelled *S. epidermidis* or *S. aureus,* respectively. At 4 °C, binding and association of bacteria with osteoblasts was seen by cytofluorometry and laser scan microscopy, respectively (Fig. 2a, b). The data were confirmed with ^3^H-thymidine-labelled bacteria. Following incubation for 3 h at 37 °C or 4 °C, cell-associated radioactivity was measured, and was found to be similar at the two temperatures. Binding could be inhibited by heparin (on average by 30 %) as reported before by others [28] (Table 1). To determine uptake of bacteria, again ^3^H-thymidine-labelled bacteria were used. Following incubation with osteoblasts for 3 h at either 4 °C or 37 °C, the cells were treated with vancomycin to kill adherent, but not internalised cells; then cell lysates were prepared and spread onto agar plates. After 24 h, bacteria colonies were counted as indicator of internalised bacteria. As seen in Table 1, internalisation occurred at 37 °C, but not at 4 °C (data summarised in Table 1).

**Table 1:** Phagocytosis of live bacteria

	bacteria associated with cells (% of input) (mean of *n* = 4)		colonies counted after 24 h	
	37 °C	4 °C	37 °C	4 °C
osteoblasts + *S. epidermidis*	47 ± 15	51 ± 21	257; 65*	52; 14*
osteoblasts + heparin + *S.epidermidis*	39 ± 21	34 ± 12	67; 38*	7; 8*

*The values represent each the mean of duplicates; two independent experiments are shown

**Fig. 3:**
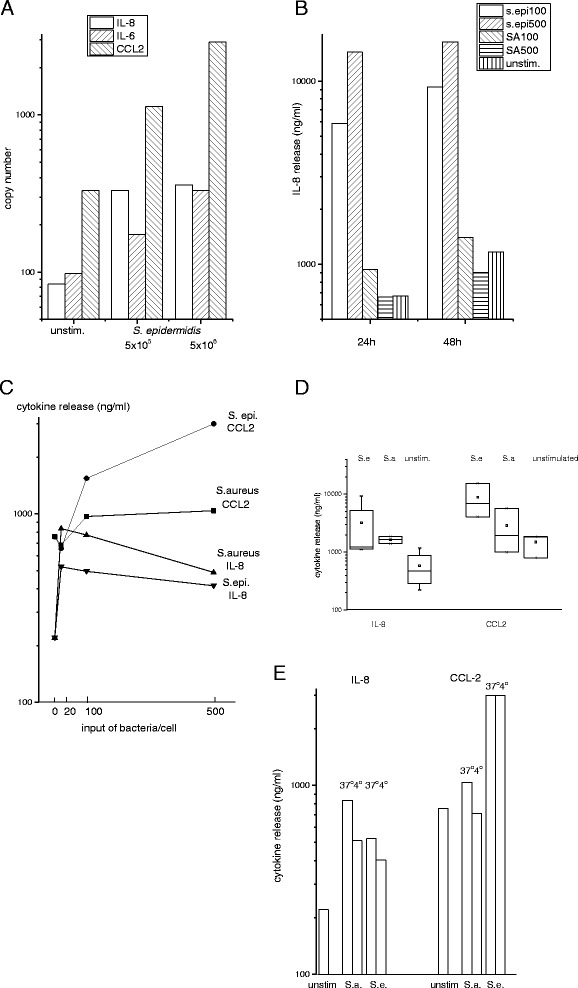
Cytokine production and release in response to bacteria: **a** Osteoblasts were cultivated with *S. epidermidis* in two concentrations and after 6 h, RNA for IL-6, IL-8, and CCL2 was quantified (data with cells of one individual are shown; mean of duplicates). Cytokine release into the supernatant was determined 24 h and 48 h after exposure to either *S. aureus* or *S. epidermidis* (bacteria to cell ratio 100 or 500 bacteria/per cell) (**b**) or (**c**) with a ratio of 1:20;100 or 500 (data with cells of one individual are shown). **d** shows the summary of experiments with cells of 5 individuals stimulated with bacteria (1:100) for 24 h. **e** osteoblasts were cultivated with bacteria (1:100) at either 4 °C or 37 °C for 20 min; after washing culture was continued for 24 h and cytokines in the supernatant were measured

In a next set of experiments effects on cytokine release in response to bacteria was measured. When osteoblasts were exposed to live bacteria, an increase in mRNA of IL-6, IL-8 and CCL2 was observed (Fig. 3a), and after prolonged incubation also release into the surrounding medium. IL-8 release was delayed compared to release of CCL2 (Fig. 3b, c). The absolute concentrations of cytokines varied widely when cells of different donors were compared. In all experiments, however, exposure to bacteria increased the concentration of IL-6, IL-8 or CCL2. On a given bacteria-to-osteoblast ratio, *S. epidermidis* apparently induced more cytokines compared to *S. aureus*, although due to the wide variation of responses, a categorical difference cannot be deduced (Fig. 3d). Of note, initial contact of osteoblasts with bacteria at 4 °C, which results in binding but not in uptake, was sufficient to induce cytokine synthesis (Fig. 3e).

### Effects of extracellular biofilm substance on osteoblasts

**Fig. 4:**
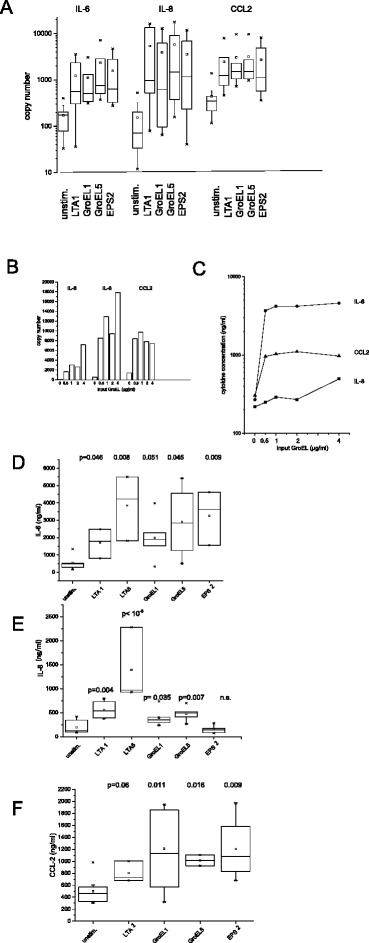
Cytokine production and release in response to bacterial biofilm EPS or EPS-derived materials: **a** Osteoblasts were cultivated for 6 h with either LTA1 (1 μg/mL), GroEL1 or GroEL5 (1 and 5 μg/mL, respectively), or EPS2 (2 % (v/v)) extracted from *S. epidermidis* biofilms; then cytokine production was determined by quantitative RT-PCR (data of 5 individuals are summarized). **b** and **c** show a dose–response curve for GroEL with cells from one individual (mean of duplicates); RT-PCR was performed after 6 h in culture, release into the supernatant after 24 h. **d**–**f** show the summary of experiments with cells of 5–7 individuals. Cytokines were measured in the cell-free supernatant after 24 h stimulation with LTA1 and LTA5 (1 and 5 μg/mL, respectively) (for CCL2, only LTA1 was measured), GroEL1 and GroEL5 (1 and 5 μg/mL, respectively) or EPS2 (2 % (v/v))

To gain information on effects of bacterial biofilms on osteoblasts, soluble entities of the extracellular polymeric substance (EPS) of biofilms were prepared. EPS consists mainly of water, a variety of glycoproteins and complex carbohydrates, lipids, and bacterial DNA. Total EPS when added in high concentration (10 % (v/v)) was toxic for the osteoblasts, lower concentrations of 2 % induced synthesis of IL-8, IL-6, and CCL2 (Fig. 4a). Precipitating proteins from the EPS revealed that both, the precipitated protein fraction, as well as the remaining non-precipitable fraction contained activating entities. A reasonable good candidate for the non-precipitable entity is lipoteichoic acid (LTA); and indeed, we could demonstrate an activation of cytokine synthesis and release by LTA (data summarised in Fig. 4a). We further tested the bacterial heat shock protein GroEL, which is present in the protein fraction of EPS. GroEL induced IL-6, IL-8 and CCL2 release within 6 to 24 h. Again, the responsiveness varied among donors, and again, release of IL-8 was somewhat prolonged compared to CCL2 (Fig. 4a–f). The effect on IL-8 induction was also small, but conspicuous when directly comparing unstimulated cells with cells exposed to GroEL derived from the same individual (data summarised in Fig. 4c).

## Discussion

Implant-associated infections are a feared complication in the field of orthopaedics, since these infections are difficult to diagnose and usually require prolonged treatment [29–31]. The reason for these difficulties is ascribed to the fact that bacteria attach to an implant surface, produce and embed themselves in an extracellular polymeric substance and are thus capable of forming biofilm-colonies that are resistant to antibiotic treatment (reviewed in [17–19, 32, 33]). Moreover, it has been proposed that bacteria in biofilms are protected from the immune system; the latter even being incapable of recognising biofilms [34–36].

However, given the fact that biofilm-formation is actually the preferred and thus probably predominant living-form of bacteria, makes it unlikely that the immune system does not respond to these infections. In support of this theory it has been shown that immune cells infiltrate areas of biofilm formation and attack biofilms [22, 37]. Of note, phagocytic cells recognize biofilms even without opsonisation; a process which is required for recognition and phagocytosis of free-swimming bacteria [38, 39]. Indeed, neutrophils were activated by the eluted extracellular matrix, and more recently, we identified lipoteichoic acid (LTA) and the bacterial heat shock protein GroEL as activating entities within the biofilm matrix [26]. In neutrophils, GroEL leads to an up-regulation of activation-associated markers on the cell surface and increased oxygen radical production [26].

Since neutrophils are not necessarily the first cells to encounter a biofilm infection, we now evaluated the effects of the extracellular polymeric substance, LTA, and GroEL on local tissue cells, namely osteoblasts. We were able to demonstrate that osteoblasts responded by an increased production of pro-inflammatory cytokines which in turn can attract immuno-competent cells, and might thus initiate or perpetuate the defence against biofilm infections. That osteoblasts produce cytokines in response to inflammatory stimuli has been reported before by others [4]; the novel aspect of our study is, that bacteria-derived entities and by implication bacterial biofilms activate the inflammatory potential of osteoblasts, most likely as the first reaction to an infection.

Another interesting aspect of osteoblast-bacteria interaction is the uptake of bacteria by osteoblasts and the intracellular survival of bacteria. In extension of previous studies by others [3, 28, 40] we found that the contact of bacteria with the osteoblast is sufficient to induce cytokine induction. Moreover, in line with data by others, this effect could be inhibited by heparin, which interacts with fibronectin-binding; the latter being essential for internalization of bacteria [28, 41]. We also demonstrated that bacteria are capable of intracellular survival in osteoblasts which might offer a feasible explanation of persisting and recurring bone infections. These findings are in line with our previous findings on enhanced cytokine expression in the tissue surrounding an infected implant which might be generated not merely by infiltrating leukocytes, but also local tissue cells [24, 42, 43].

## Conclusion

In conclusion, we were able to show that aside from the obvious role of osteoblasts in bone formation, they also respond to bacterial infections. Osteoblasts are capable of increased expression and release of pro-inflammatory cytokines when challenged with free-swimming bacteria. Furthermore, we demonstrated internalization of bacteria and intracellular survival as a method of bacterial persistence leading to chronic infectious disease.

Osteoblasts not only respond to free-swimming bacteria, but also to components of the extracellular polymeric substance, among them the bacterial heat shock protein GroEL, and may therefore be critically involved in defence mechanisms against biofilm infections.

## Abbreviations

CCL2, monocyte chemotactic protein 1 (MCP1α); EPS, extracellular polymeric substance; IL-6, Interleukin-6; IL-8, Interleukin-8; LTA, lipoteichoic acid; qRT-PCR, quantitative real-time polymerase chain reaction.
